# Primary Diffuse Large B-Cell Lymphoma of the Urinary Bladder: Update on a Rare Disease and Potential Diagnostic Pitfalls

**DOI:** 10.3390/curroncol29020081

**Published:** 2022-02-10

**Authors:** Magda Zanelli, Francesca Sanguedolce, Maurizio Zizzo, Andrea Palicelli, David Pellegrini, Sabrina Farinacci, Alessandra Soriano, Elisabetta Froio, Luigi Cormio, Giuseppe Carrieri, Alberto Cavazza, Francesco Merli, Stefano A. Pileri, Stefano Ascani

**Affiliations:** 1 Pathology Unit, Azienda USL-IRCCS di Reggio Emilia, 42123 Reggio Emilia, Italy; andrea.palicelli@ausl.re.it (A.P.); elisabetta.froio@ausl.re.it (E.F.); alberto.cavazza@ausl.re.it (A.C.); 2 Pathology Unit, Policlinico Riuniti, University of Foggia, 71122 Foggia, Italy; francesca.sanguedolce@unifg.it; 3 Surgical Oncology Unit, Azienda USL-IRCCS di Reggio Emilia, 42123 Reggio Emilia, Italy; maurizio.zizzo@ausl.re.it; 4 Pathology Unit, Azienda Ospedaliera Santa Maria di Terni, University of Perugia, 05100 Terni, Italy; d.pellegrini@aospterni.it (D.P.); s.farinacci@aospterni.it (S.F.); s.ascani@aospterni.it (S.A.); 5 Gastroenterology Division, Azienda USL-IRCCS di Reggio Emilia, 42123 Reggio Emilia, Italy; alessandra.soriano@ausl.re.it; 6 Department of Urology and Renal Transplantation, University of Foggia, 71122 Foggia, Italy; luigi.cormio@unifg.it (L.C.); giuseppe.carrieri@unifg.it (G.C.); 7 Hematology Unit, Azienda USL-IRCCS di Reggio Emilia, 42123 Reggio Emilia, Italy; francesco.merli@ausl.re.it; 8 Haematopathology Division, European Institute of Oncology-IEO IRCCS Milan, 20141 Milan, Italy; stefano.pileri@unibo.it

**Keywords:** diffuse large B-cell lymphoma, urinary bladder, extranodal, lymphoma

## Abstract

Diffuse large B-cell lymphoma (DLBCL) represents the most frequent type of non-Hodgkin lymphoma. Globally, DLBCL is an aggressive disease, requiring an accurate diagnosis and prompt treatment. The diagnosis is often made on biopsy samples of a nodal mass, however, approximately 40% of DLBCL cases arise at extranodal sites. The most common extranodal site is the gastrointestinal tract, however any extranodal area may be primarily involved. Primary urinary bladder lymphoma represents only 0.2% of extranodal non-Hodgkin lymphomas, whereas secondary involvement of the urinary bladder by a systemic lymphoma is a more common event. Despite being rare, DLBCL is considered to represent the predominant primary urinary bladder lymphoma. The majority of cases reported in the bladder belong to the DLBCL, NOS group, and there are only rare cases of EBV-positive DLBCL, NOS. In this review, we summarize the current knowledge on DLBCL primarily occurring in the urinary bladder, with the aim of increasing clinician and pathologist awareness on this aggressive lymphoma rarely arising in the urinary bladder. Additionally, we focus on those entities which should be taken into consideration in the differential diagnosis, highlighting potential diagnostic pitfalls.

## 1. Introduction

Non-Hodgkin lymphomas (NHLs) are the most frequent hematological neoplasms. Diffuse large B-cell lymphoma (DLBCL) accounts for 30–40% of NHLs, being the most common NHL subtype [1].

DLBCL encompasses a broad and heterogeneous group of variants, recognized in the current WHO classification, with distinctive morphological, immunohistochemical or genetic features and distinctive biological behavior [1]. Diffuse large B-cell lymphoma not otherwise specified (DLBCL-NOS) does not belong to any known, specific variant of DLBCL, hence being such named. The usual clinical presentation is with a rapidly growing nodal or extranodal mass. In particular, extranodal sites represent the primary site of DLBCL occurrence in about 40% of cases and the disease may be limited to these locations at least initially [1].

Extranodal lymphomas account for 25–35% of all NHLs and may arise in almost any organ [1,2].

Primary urinary tract lymphomas (PUTLs) represent less than 5% of all extranodal NHLs [2,3,4]. Primary urinary bladder (UB) lymphoma is a very rare disease, representing less than 0.2% of all extranodal lymphomas and less than 1% of all UB neoplasms [2,3,4,5,6,7,8,9,10,11,12,13,14]. Secondary involvement of the UB by systemic lymphoma is a much more common event, being identified in 10–20% of NHLs [11]. 

Most cases of DLBCL of the urinary tract (UT) arise from the kidney (72.39%), followed by the UB (24.95%) [1,14]. Extranodal marginal zone B-cell lymphoma of mucosa-associated lymphoid tissue (MALT lymphoma) has long been considered the most common primary UB lymphoma [15], until, recently, in a large study on PUTL, DLBCL has been recognized to be the predominant type of primary UB lymphoma [8]. DLBCL is generally an aggressive disease requiring a prompt therapy. If not adequately treated, it leaves the patients a prognosis of a few months. For the early recognition and treatment of this neoplasm, it is critical for urologists and pathologists to be aware that this disease may arise even in the UT, including the UB. To the best of our knowledge, mainly isolated case reports and small case series of primary UB-DLBCL have been published so far. This review aims to make a comprehensive and detailed overview of the clinicopathological features of primary UB-DLBCL, focusing on differential diagnoses and potential diagnostic pitfalls to be taken into consideration. The therapeutic strategies adopted in this setting of patients are also discussed.

## 2. General Overview on Primary Lymphomas of the Urinary Tract

PUTL, first described by Jacobs and Symington in 1953, represents a rare disease [3]. The large-scale population study by Lontos et al. evaluated 1264 cases of PUTL which had been diagnosed in the USA in the period between 1983 and 2013.

Only 24% of cases occurred in patients less than 60 years of age and the median age at presentation was 71 years [8]. PUTL shows a slight male predominance (57%), and the kidney is the site most frequently involved (70.8%) [8]. At presentation, the disease is more often in stage 1 (40% of cases), although 32% of cases are detected in stage IV. 

The most common histological type of PUTLs is DLBCL (51.9%), followed by MALT lymphoma (12.5%) and follicular lymphoma (9.4%). According to the study by Lontos et al., UB lymphoma is prevalent in females (58.7%), whereas renal lymphoma prevails in males (63.3%). MALT lymphoma is more frequent in the UB (22.7%) than in the kidney (12.7%). The higher frequency of MALT lymphoma in the UB as well as the observation that UB lymphomas are more frequent in women supports the hypothesis that recurrent infectious cystitis, more common in females, represents an event favoring the occurrence of MALT lymphoma [8,12]. 

UB lymphomas more often present with recurrent urinary tract infections and hematuria, whereas renal lymphomas usually present with fever, weight loss, abdominal or flank pain, and hematuria [8,12]. Most UB lymphomas are diagnosed at stage 1 (61.3%) compared to 31.4% of kidney lymphomas. The possible reason for this difference between the two sites may be the faster presentation with hematuria of UB lymphomas. 

Extranodal DLBCL often carries a worse outcome compared to the nodal counterpart [16]. DLBCL arising in the UT has been found as well to have a worse prognosis compared to primary nodal DLBCL in both early and advanced stages of the disease [8]. Five-year cancer specific survival (CSS) is better in early-stage nodal DLBCL (69%) than in UT-DLBCL (59%); similarly in late-stage disease, UT-DLBCL shows a 5-year CSS (39%) worse than nodal DLBCL (46%). Poor prognostic factors in PUTL are late-stage disease, DLBCL histology, male gender, and older age [8].

## 3. Primary DLBCL of the Urinary Bladder: Clinical Features

Although Kempton et al. as well as others stated that the most common type of primary UB lymphoma is MALT lymphoma (44.4%) followed by DLBCL in 20% of cases [12,15], in the recent study by Lontos et al., DLBCL was recognized to be the predominant histology comprising 60.3% of primary UB lymphoma, whereas MALT lymphoma was identified in 22.7% of UB lymphoma cases [8]. The study by Liu et al., evaluating 489 patients with UT-DLBCL diagnosed between 1975 and 2016, is the first study analyzing the clinical features and survival outcomes for UT-DLBCL in a large population [14]. Liu et al. found that the majority of UT-DLBCL originates from the kidney (72.39% of cases), followed by the UB (24.95% of cases) [14]. 

Patients over 60 years are more likely to be affected by UT-DLBCL, with a mean age of 69 years at diagnosis. Patients with kidney DLBCL are younger (68 years) than those with UB-DLBCL (76 years). The increase in UT-DLBCL with aging is likely to be due to the decline of the immune system in older individuals, which is known as immune senescence; chronic inflammation, which increases with aging, is an additional factor predisposing to lymphoma development. 

Kidney DLCL is the most frequent form in males (59.89%) and UB-DLBCL in females (59.84%)

Hematuria is the most common presenting symptom of UB-DLBCL; other symptoms are urinary frequency, dysuria, nocturia, pain in the lower abdomen, and frequent UT infections. 

Most UB-DLBCL (56.56%) are diagnosed as stage 1, compared with only 25.71% of kidney DLBCL cases. Older age represents a poor prognostic factor in UT lymphomas in general [8], and in UT-DLBCL as well, the mortality of patients over 75 years is 2–3 times higher than that of younger patients [14], with a 5-year overall survival (OS) of 27.10% for patients over 75 years compared to 64.29% for patients under 60 years. 

## 4. Diagnostic Approach for Primary Urinary Bladder Lymphoma 

On the basis of nonspecific lower urinary tract symptoms (LUTS), the initial clinical impression for primary UB lymphoma may be of UT infection or urothelial carcinoma. A delay in diagnosis and treatment of UB lymphoma may be caused by the rather nonspecific symptomatology. Diagnosing a lymphoma in the UB relies on imaging techniques, cystoscopy, and a complete pathologic evaluation of UB biopsies.

**Figure 1:**
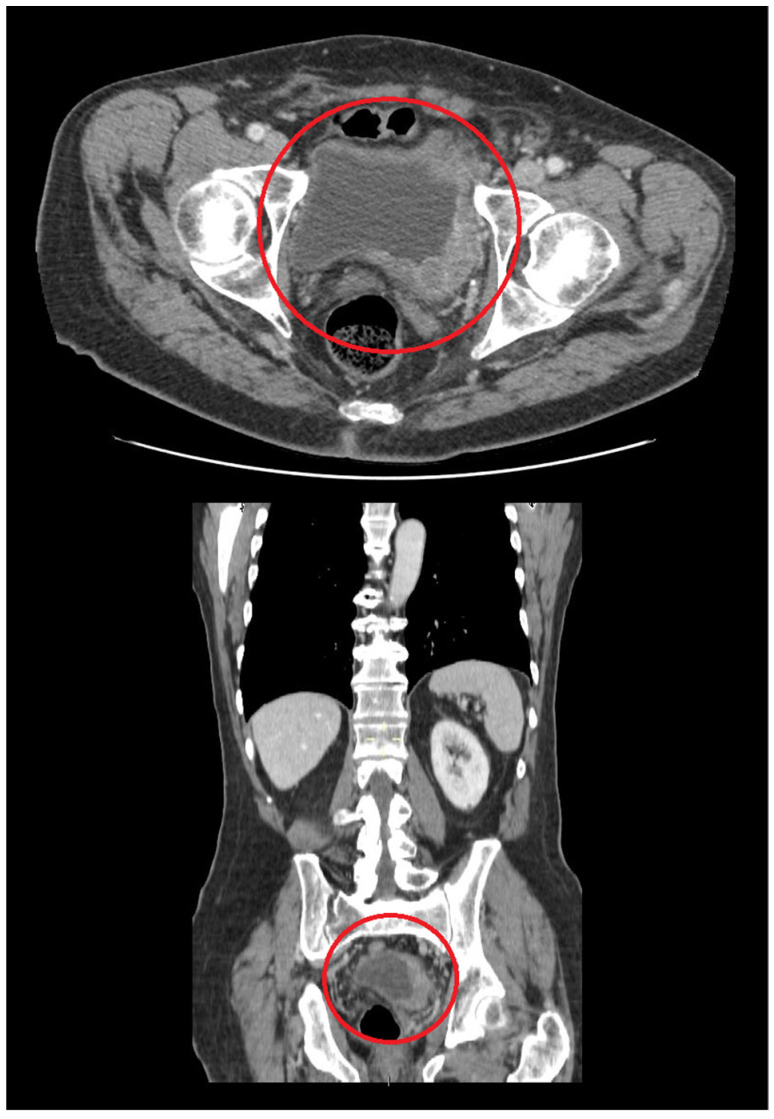
CT scan showing a large urinary bladder mass (within the red circle), histologically confirmed to be a primary EBV-positive DLBCL, NOS (original image from Dr. M. Zizzo; unpublished case).

Imaging techniques such as ultrasound, computed tomography (CT) scan (Figure 1), and magnetic resonance imaging (MRI) can provide detailed information about size and localization of any tumor in the UT, including the UB. CT and MRI features do not allow to suspect UB carcinoma rather than UB lymphoma. Cystoscopy and histological evaluation of biopsy samples play a major role in the diagnostic work-up of patients with a UB neoplasm. In particular, the diagnosis of UB lymphoma needs the histological evaluation of the tumor tissue with proper ancillary techniques such as immunohistochemical stainings and sometimes molecular testing.

When a diagnosis of DLBCL is made on UB biopsies, staging procedures are essential in order to establish if UB involvement is secondary to a systemic lymphoma, which is a more common event, or if the UB itself represents the primary site of disease occurrence. 

The definition of primary versus secondary lymphoma involvement of extranodal sites such as the UB may be problematic. Following the criteria proposed by Krol et al., any lymphoma initially presenting at an extranodal site should be considered extranodal; similarly, in cases of disseminated diseases, if the extranodal component is clinically dominant, the lymphoma should be considered extranodal [17]. Due to its higher sensitivity, in the context of DLBCL, 18F-fluorodeoxyglucose positron-emission tomography with computed tomography (PET-CT) replaced CT scan for staging as well as for evaluating end of therapy response.

## 5. Histology, Immunophenotype, and Genetic Features of DLBCL, NOS

DLBCL represents a heterogeneous category and, in the current WHO classification, under the broad heading of DLBCL, there are several entities with distinct clinicopathological and biologic features [1,18,19]. 

DLBCL, NOS is the most common category of DLBCL, representing about 80–85% of cases [1]. Histologically, DLBCL, NOS consists of a diffuse proliferation of large-sized cells effacing the architecture of the involved tissue. Neoplastic cells resemble either centroblasts (CBs) in 80% of cases or immunoblasts (IBs) in about 10% of cases. In rare cases of the so-called anaplastic variant, the cells have a bizarre, pleomorphic appearance. 

Neoplastic elements are positive for pan B-cell markers (CD20, CD79 alpha, PAX5, CD22, and CD19) and CD45 and, usually, express surface immunoglobulin. 

Based on gene expression profiling (GEP), DLBCL is classified into distinct prognostic subgroups as follows: the germinal center B-cell (GCB)-like subtype (40–50% of cases), the activated B-cell (ABC)-like subtype (50–60%) and unclassified subtype (10–15%) [20,21]. DLBCL of GCB subtype shows a gene signature characteristic of normal germinal center B cells with CD10 and BCL6 expression, hypermutated immunoglobulin, and ongoing somatic hypermutation. DLBCL of ABC subtype has a gene signature of post-germinal center B cells with expression of MUM1/IRF4 and nuclear factor kappa B (NF-kB) activation. The cell of origin (COO) classification identifies distinct prognostic subgroups with the ABC subtype being associated with a worse outcome compared to the GCB subtype. The introduction of rituximab in DLBCL treatment has reduced the prognostic impact of COO classification, despite remaining the ABC subgroup less responsive to therapy [22,23,24,25]. 

Despite the prognostic significance of the GEP-based classification, the need for fresh or frozen (FF) samples makes GEP not easily applicable in routine daily practice; hence, several immunohistochemistry (IHC) algorithms have been proposed as a surrogate for GEP analysis [1,26,27,28]. The Hans and Tally algorithm, based on the expression of CD10, BCL6, and MUM1/IRF4, is the widest method applied [1,26]. However, the rate of concordance between GEP and immunohistochemical algorithms is variable (65–90%) and, in particular, subjectivity in immunohistochemical result interpretation, as well as variability in the immunohistochemical techniques performed, makes immunohistochemistry-based algorithms not completely reliable. 

Subsequently, customized GEP mostly applied on the NanoString platform to formalin-fixed, paraffin-embedded (FFPE) samples has been found to represent a more reliable technique for predicting prognosis compared to the immunohistochemical algorithms [29]. 

In a subset of B-cell lymphomas, which, based on morphology and phenotype, would be regarded as DLBCLs-NOS, genetic rearrangements of *C-MYC*, *BCL6*, and *BCL2* have been identified by fluorescent in situ hybridization (FISH) analysis. These lymphomas are known as double-hit (DH) or triple-hit (TH) lymphomas and, in the 2017 WHO classification, they belong to the provisional category of High-grade B-cell lymphomas with *MYC* and *BCL2* or *BCL6* rearrangements, or both (HGBCL-DH/TH) [1]. This category shows a worse outcome and may require more intense chemotherapeutic strategies than standard R-CHOP regimen. In order to identify this more aggressive subset of lymphomas, FISH analysis should be applied to all DLBCL-NOS cases. 

New insights into DLBCL pathogenesis have been recently provided by high-throughput techniques identifying genetic subtypes of DLBCL with distinct clinical behavior [30].

A total of four main genetic subtypes of DLBCL have been identified by Schmitz et al.; they have been designated N1 (on the basis of *NOTCH1* mutations), EZB (on the basis of *EZH2* mutations and *BCL2* translocations), MCD (because of the co-existence of *MYD88* and *CD79B* mutations), and BN2 (based on *BCL6* fusions and *NOTCH2* mutations) [30].

The BN2 and ENZ subtypes showed a better prognosis compared to the MCD and N! subtypes [30].

## 6. EBV-Positive DLBCL, NOS and Primary Bladder Lymphoma

Of the DLBCL cases primarily occurring in the UB, the majority are DLBCL, NOS, with only two cases of EBV-positive DLBCL, NOS reported so far [31,32].

EBV-positive DLBCL, NOS was initially described in patients older than 50 years and, hence, named EBV-positive DLBCL of the elderly in the 2008 WHO classification [33,34]. The terminology has been changed in the current WHO classification as the disease has been identified even in individuals younger than 50 years [1,35,36]. EBV-positive DLBCL, NOS may arise at nodal and extra-nodal sites. In younger patients the disease more often arises at nodal sites, presenting at lower stage and with a better outcome [35,36]. In older individuals the disease more often occurs at extranodal sites, following a worse prognosis [37]. 

Among the extranodal sites, skin, lung, and gastrointestinal tract (GIT) are more often involved [37,38,39,40]. 

One of the two cases of EBV-positive DLBCL, NOS arising in the bladder occurred in a patient undergoing treatment for prostate cancer with enzalutamide and lymphoma spontaneously regressed after the cessation of this therapy [32]. This latter case would probably be reclassified as iatrogenic immunodeficiency-associated lymphoproliferative disorder (LPD), according to the current WHO classification [1].

**Figure 2:**
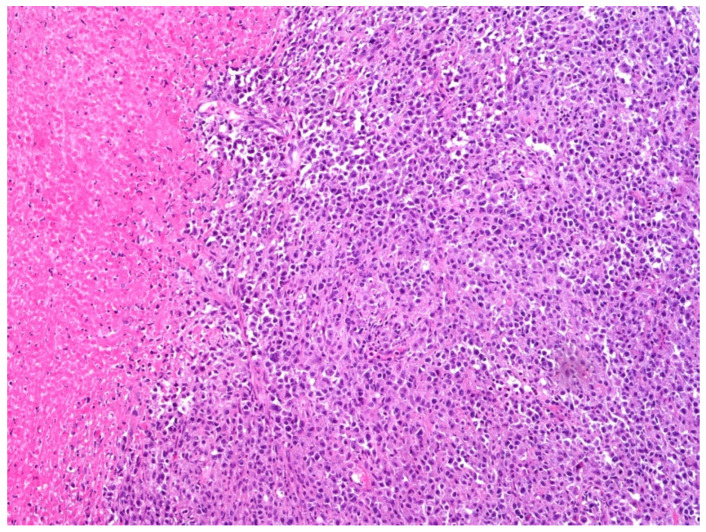
EBV-positive DLBCL, NOS composed of sheets of large-sized cells admixed with necrotic foci (hematoxylin and eosin, 200× magnification; original histological image from Dr. M. Zanelli; unpublished case).

**Figure 3:**
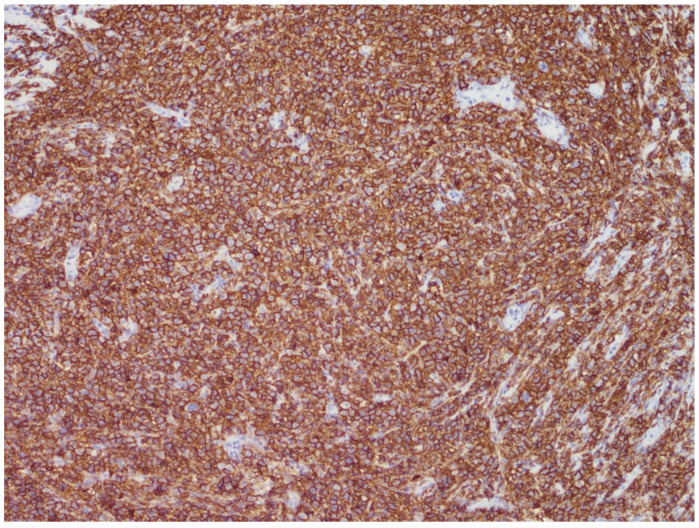
EBV-positive DLBCL, NOS: CD20 immunostaining highlighting the B-cell phenotype of the neoplastic cells (immunostain, 200× magnification; original histological image from Dr. M. Zanelli; unpublished case).

**Figure 4:**
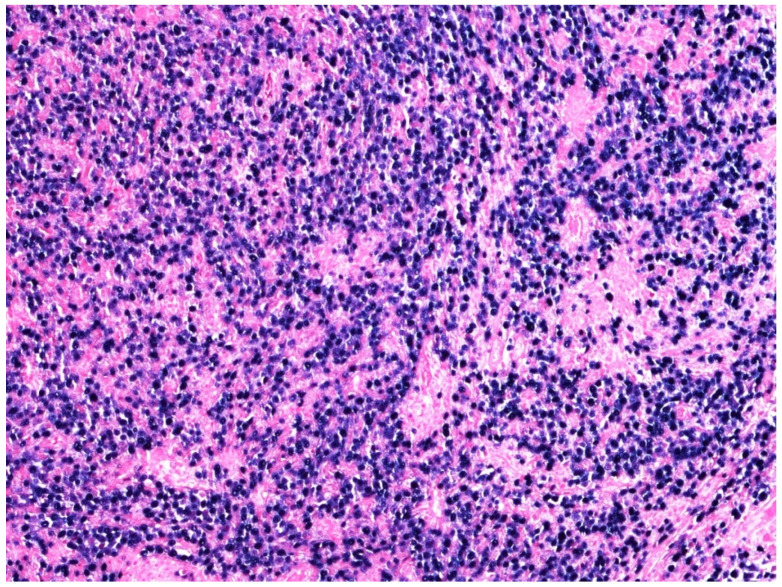
EBV-positive DLBCL, NOS: neoplastic cells diffusely EBER positive (in situ hybridization, 200× magnification original histological image from Dr. M. Zanelli; unpublished case).

A total of two morphological patterns are recognized in EBV-positive DLBCL, NOS: the monomorphic pattern and the polymorphic pattern. The former is composed of sheets of large-sized cells (Figure 2 and Figure 3) and is indistinguishable from the EBV-negative counterpart of DLBCL, NOS, unless in situ hybridization for EBV-encoded RNA (EBER) is performed (Figure 4).

The polymorphic or T-cell/histiocyte-rich large B-cell lymphoma-like pattern consists of scattered large-sized cells often resembling Hodgkin and Reed–Sternberg cells, admixed with inflammatory elements. Necrosis is often found. Of the two reported cases of EBV-positive DLBCL, NOS primarily arising in the UB, one showed a polymorphic pattern [31].

Overall, compared with the EBV-negative form, EBV-positive DLBCL, NOS more often shows a non-GCB phenotype. CD30 is often expressed with rare CD15 positivity. EBER expression in the majority (about 80%) of neoplastic cells is required for diagnosis [1,34]. Type II or type III latency pattern are usually observed in the disease, LMP1 expression being found in 2/3 of cases and EBNA2 in 1/3 of cases.

The genetic profile of EBV-positive DLBCL, NOS is different from its negative counterpart. In EBV-positive DLBCL, NOS, EBV itself favors the activation of the Janus kinase signal-transducer and activator of transcription (JAK-STAT) and NF-Kappa B pathways [41]. In addition, in the EBV-positive form, chromosomal gains at 9p24.1 contribute to increasing the expression of programmed cell death ligand 1 and 2 (PD-L1 and PD-L2), therefore, favoring immune tolerance and tumor evasion [37]. 

## 7. Differential Diagnoses and Potential Diagnostic Pitfalls

In the UB, DLBCL may be potentially misdiagnosed as poorly differentiated carcinoma; other neoplastic entities to be ruled out are melanoma and aggressive B-cell lymphomas typically arising at extranodal sites such as plasmablastic lymphoma (PBL), the extracavitary variant of primary effusion lymphoma (EC-PEL), and Burkitt lymphoma (BL). Table S1 summarizes the clinicopathological features of aggressive B-cell lymphomas primarily occurring in the UB (Supplementary Materials).

A comprehensive immunohistochemical panel is essential for a precise and accurate diagnosis. 

S100 protein and other melanoma markers (HMB-45, Melan-A/MART-1, and SOX10) clearly establish a UB melanoma diagnosis. It is worth mentioning that MUM-1/IRF4, a known lymphocyte markers often expressed in DLBCL, may be positive in melanocytic lesions [42]. 

Cytokeratin immunohistochemical expression is essential for distinguishing urothelial carcinoma and DLBCL [43]. Lymphoepithelioma-like carcinoma of the bladder is a type of bladder cancer with a rather characteristic histology. The neoplastic cells show a syncytial pattern of growth within a background rich in inflammatory cells [44]. The histological appearance of lymphoepithelioma-like carcinoma may resemble the polymorphic pattern of EBV-positive DLBCL. Unlike nasopharyngeal lymphoepithelioma-like carcinoma, which is strongly associated with EBV infection, UB lymphoepithelioma-like carcinoma does not appear to be associated with EBV [45].

Additionally, it must be kept in mind that the possible expression of epithelial markers by lymphoma cells as a potential pitfall in the differential diagnosis between poorly differentiated carcinoma and DLBCL. P63 has a role in the development and differentiation of stratified epithelia, and it is expressed in normal tissues, including urothelium and squamous epithelium [46]. Therefore, p63 is often interpreted as a marker of epithelial differentiation and carcinoma. Urothelial carcinoma is frequently p63 positive. 

Expression of p63 is observed in a significant number of DLBCL cases, with variable percentage (from 15% to 70% of cases) depending upon the different studies [47,48,49]. Hence, p63 expression alone does not rule out a lymphoma diagnosis. 

GATA binding protein 3 (GATA-3) is another marker which may represent a potential source of misdiagnosis between carcinoma and lymphoma. GATA-3 is commonly used in the diagnostic work-up of urothelial and breast carcinomas, with a high sensitivity in these neoplasms [50]. Little is known about GATA-3 expression in lymphomas. Despite being unreported in DLBCL so far, GATA-3 positivity is observed in classic Hodgkin lymphoma (cHL) [51] and in a subset of peripheral T-cell lymphoma; in particular, GATA-3 expression correlates with poor prognosis in peripheral T-cell lymphoma [52].

PBL should be taken into consideration in the differential diagnosis, due to its common extranodal presentation [1,53,54,55,56,57,58,59]. PBL is an aggressive lymphoma usually diagnosed in HIV-positive individuals, but it also occurs in other settings of immunosuppression such as organ transplantation, iatrogenic-immunosuppression, or autoimmune disorders. PBL has been initially reported in the oral cavity in HIV-positive individuals, but GIT and skin are other sites commonly involved. PBL arising in the UT in general or in the UB is very unusual [60,61]. Histologically, PBL consists of sheets of IBs and plasmablasts (PBs) usually expressing plasma cell-associated markers such as CD138, CD38, and MUM1/IRF4; unlike in DLBCL, NOS, CD20 is often not expressed in PBL. EBER is positive in the majority of PBL, often showing a type I latency pattern, whereas in EBV-postive DLBCL, NOS, there is often a predominance of type II and type III latency pattern. Unlike EBV-positive DLBCL, which usually lacks *MYC* translocation, PBL often shows *IGH/MYC* translocation.

EC-PEL should be taken into consideration in the differential diagnosis with EBV-positive DLBCL. PEL is a high-grade B-cell neoplasm usually arising in immunocompromised individuals, often HIV patients or older individuals [62,63]. PEL belongs to the spectrum of HHV8-associated diseases [1,64]. Whereas the classic variant of PEL is a body cavity-based lymphoma presenting with effusions, the EC variant presents with solid masses [65,66]. EC-PEL may occur in lymph nodes, GIT, the central nervous system, and skin [65,66,67,68,69,70]. As far as we are concerned, only one case of EC-PEL occurring in the UB and ureter has been reported so far [71]. In contrast with PEL in its classic form, which is often negative for B-cell markers, the EC variant is more commonly positive for at least some B-cell markers, making the differential diagnosis with DLBCL, NOS more difficult. The expression of Kaposi Sarcoma Herpes Virus/Human Herpes Virus 8 (KSHV/HHV8) is a required criterion for PEL diagnosis. In PEL occurring in HIV-positive individuals, there is usually a co-infection of HHV8 and EBV, whereas in HIV-negative individuals, PEL is often EBV negative [1].

BL is another highly aggressive B-cell lymphoma usually arising at extranodal sites [1,72].

Its primary occurrence in the UB has been rarely reported [73,74]. BL is often linked with EBV infection, although the rate of association with EBV varies according to the epidemiological subtypes of BL; in the endemic variant in particular, EBV is detected in the majority of cases. It consists of a diffuse proliferation of highly proliferating medium-sized cells characterized by a rather striking monomorphism. The typical starry sky pattern is indicative of the high proliferation rate of this lymphoma. BL has a B-cell phenotype, with expression of germinal center markers (BCL6 and CD10), and usually absence of BCL2. In most BL cases, the reciprocal chromosomal translocation between the *MYC* proto-oncogene and one of the immunoglobulin genes is generally present.

BL enters in the differential diagnosis with HGBCL-DH/TH with *MYC* and *BCL2* or *BCL6* rearrangements, or both. This group of lymphomas may histologically resemble BL or show features in between BL and DLBCL. The often strong BCL2 expression observed in HGBCL-DH/TH contrasts with the absent or weak BCL2 expression in BL.

## 8. Treatment of Primary UB-DLBCL

The use of chemo-immunotherapy usually with R-CHOP (rituximab, cyclophosphamide, doxorubicin, vincristine, and prednisone) scheme represents the mainstay of nodal DLBCL lymphoma treatment [75], and it is used in extranodal DLBCL as well. In the study by Liu et al., patients not undergoing chemo-immunotherapy had about twice the risk of death compared to patients receiving the treatment [14].

Unlike patients with nodal DLBCL obtaining benefit from radiation therapy, patients with UT-DLBCL had no survival benefits with radiotherapy.

Surgery resulted in being beneficial, especially for kidney DLBCL, whereas no beneficial effect was seen on patients with UB-DLBCL, possibly because of the morbidity and mortality associated with surgery in UB lymphomas.

In general, the category of DLBCLs can be cured with the R-CHOP regimen in more than 60% of cases and new therapeutic approaches have been used in cases in which R-CHOP failed [76]. Because of the rarity of UT-DLBCL, further studies are essential in order to better define the benefit of immunochemotherapy in terms of survival.

As a whole, the prognosis of patients with EBV-positive DLBCL, NOS is worse compared with patients with the EBV-negative counterpart [77]. Currently, in different types of tumors, the immune evasion by PD-L1 and PD-1 pathways represents a potential therapeutic target [78]. Compared to EBV-negative DLBCL, NOS, in the EBV-positive counterpart there is a higher rate of PD-L1 expression on neoplastic cells and cells of the microenvironment such as macrophages and dendritic cells [79,80]. Therefore, despite the R-CHOP regimen being used even in EBV-positive DLBCL, NOS, immunomodulatory therapies, targeting the axis PD-1/PD-L1 and drugs targeting the JAK-STAT and NF-κ pathways may represent attractive therapeutic options [36].

## 9. Conclusions

Owing to its rarity, to date, studies on the clinicopathological characteristics of DLBCL primarily arising in the UB are still limited. Urologist and pathologist awareness of the possibility that DLBCL may arise in the UB is critical as the early recognition of the disease allows a prompt and adequate treatment of an aggressive, but potentially curable lymphoma.

## Data Availability

Individual patient data from the original studies included in the present review are not available and data sharing at this level is not applicable for a systematic review.

## Supplementary Materials

The following are available online at https://www.mdpi.com/article/10.3390/curroncol29020081/s1, Table S1: Aggressive lymphomas of B-cell origin primarily affecting the UB.
