# Supplementary material for: Primary Diffuse Large B-Cell Lymphoma of the Urinary Bladder: Update on a Rare Disease and Potential Diagnostic Pitfalls
Source: Curr Oncol. 2022 Feb 10;29(2):956–68. doi: 10.3390/curroncol29020081 (PMC8870454; doi:10.3390/curroncol29020081)
Supplement: Supplementary file 1 [file curroncol-29-00081-s001.zip › curroncol-1560667-supplementary.pdf]

Current Oncology

Supplementary

Primary Diffuse Large B-Cell Lymphoma of the Urinary Bladder: Update on a Rare Disease and Potential Diagnostic Pitfalls

Magda Zanelli, Francesca Sanguedolce, Maurizio Zizzo, Andrea Palicelli, David Pellegrini, Sabrina Farinacci, Alessandra Soriano, Elisabetta Froio, Luigi Cormio, Giuseppe Carrieri, Alberto Cavazza, Francesco Merli, Stefano A. Pileri and Stefano Ascani

Table 1. Aggressive lymphomas of B-cell origin primarily affecting the UB.

	IS	Localization	UB	Histology	EBER; HHV8	Genetic profile	Prognosis
DLBCL,NOS	Rarely present	Nodal and extranodal	60.3% of primary UB lymphoma	Sheets of CBs or IBs	EBER -; HHV8 -	Monoclonal (IG gene rearrangement)	Potentially curable
EBV+ DLBCL, NOS	Rarely present	Nodal (often in young) and extranodal sites (old patients)	Rare	2 patterns of growth: Polymorphic or monomorphic	EBER +; HHV8 -	Monoclonal (IG gene rearrangement)	Poor
PBL	Mostly present (often HIV+)	Extranodal sites mainly; nodal sites rarely	Rare	PBs, IBs (diffuse pattern of growth)	EBER + in HIV+ pts and PT pts; HHV8 -	Monoclonal (IG gene rearrangement); *MYC* translocation (half of cases)	Poor
PEL	Mostly present (often HIV+)	Classic PEL: serous cavities. EC-PEL: nodal sites mostly; extranodal sites rarely	Rare (EC-PEL)	PBs, IBs (diffuse pattern of growth in EC-PEL; in fluids in classic PEL)	EBER + (- in elderly HIV-negative pts); HHV8 +	Monoclonal (IG genes hypermutated)	Poor
BL	Present in the immunodef.	Extranodal sites mainly; nodal sites in the Immunodef.	Rare	Medium-sized cells (diffuse monotonous pattern of growth)	EBER+ in >95% end.; 20–30% spor.; 25–40% immunodef. HHV8-	Monoclonal (IG gene rearrangement); *MYC* translocation (the majority of cases);	Highly aggressive, but potentially curable

Legend: BL: Burkitt lymphoma; CBs: centroblasts; DLBCL: diffuse large B-cell lymphoma; EBER: in situ hybridization for EBV-encoded RNA; EBV: Epstein-Barr virus; EBV+ DLBCL, NOS: EBV-positive, diffuse large B-cell lymphoma, not otherwise specified; EC-PEL: extra-cavitary primary effusion lymphoma; end: endemic variant; HHV8: Human herpes virus 8; HHV8+ HIV: human immunodeficiency virus; IBs: immunoblasts; Immunodef: immunodeficiency-associated variant; IG immunoglobulin; IS: immunosuppression; PBL: plasmablastic lymphoma; PBs: plasmablasts; PEL: primary effusion lymphoma; spor: sporadic variant; UB: urinary bladder.
